# The Role of Ferroptosis in the Damage of Human Proximal Tubule Epithelial Cells Caused by Perfluorooctane Sulfonate

**DOI:** 10.3390/toxics10080436

**Published:** 2022-07-29

**Authors:** Pingwei Wang, Dongge Liu, Shuqi Yan, Yujun Liang, Jiajing Cui, Li Guo, Shuping Ren, Peng Chen

**Affiliations:** 1 Department of Occupational and Environmental Health, School of Public Health, Jilin University, Changchun 130021, China; wangpw20@mails.jlu.edu.cn (P.W.); liudg21@mails.jlu.edu.cn (D.L.); yansq21@mails.jlu.edu.cn (S.Y.); liangyj20@mails.jlu.edu.cn (Y.L.); cuijl20@mails.jlu.edu.cn (J.C.); rensp@jlu.edu.cn (S.R.); 2 Department of Toxicology, School of Public Health, Jilin University, Changchun 130021, China; gli@jlu.edu.cn; 3 Department of Pediatrics, The Second Hospital of Jilin University, Changchun 130041, China

**Keywords:** perfluorooctane sulfonate, PFOS, kidney, ferroptosis, HK-2 cells

## Abstract

Perfluorooctane sulfonate (PFOS) is a typical persistent organic pollutant and environmental endocrine disruptor that has been shown to be associated with the development of many diseases; it poses a considerable threat to the ecological environment and to human health. PFOS is known to cause damage to renal cells; however, studies of PFOS-induced ferroptosis in cells have not been reported. We used the CCK-8 method to detect cell viability, flow cytometry and immunofluorescence methods to detect ROS levels and Western blot to detect ferroptosis, endoplasmic reticulum stress, antioxidant and apoptosis-related proteins. In our study, we found that PFOS could induce the onset of ferroptosis in HK-2 cells with decreased GPx4 expression and elevated ACSL4 and FTH1 expression, which are hallmarks for the development of ferroptosis. In addition, PFOS-induced ferroptosis in HK-2 cells could be reversed by Fer-1. We also found that endoplasmic reticulum stress and its mediated apoptotic mechanism and P53-mediated antioxidant mechanism are involved in the toxic damage of cells by PFOS. In this paper, we demonstrated for the first time that PFOS can induce ferroptosis in HK-2 cells. In addition, we preliminarily explored other mechanisms of cytotoxic damage by PFOS, which provides a new idea to study the toxicity of PFOS as well as the damage to the kidney and its mechanism.

## 1. Introduction

Perfluorooctane sulfonate (PFOS) is an anthropogenic chemical that falls into the category of chemicals called per- and polyfluoroalkyl substances (PFAS); it consists of a hydrophobic carbon–fluorine (CF) chain and a hydrophilic functional group (sulfonate for PFOS) [1]. The carbon–fluorine bond has one of the highest bond dissociation energies in any chemical bonds and is firmly stable. Because of the high solubility in water and high chemical stability, it is widely found on land and in water [2].

PFOS has been identified as a persistent organic pollutant (POP) and was listed into Annex B of the Stockholm Convention on Persistent Organic Pollutants [3]. The half-life of PFOS in human is 5.4 years [4]. PFOS can cause endocrine disruption in the body, thus it is considered to be one of the environmental endocrine disruptors and has been shown to be associated with the development of various diseases, such as immunotoxicity, kidney damage, liver damage, developmental toxicity, reproductive toxicity, insulin resistance and endocrine disrupting effects [5,6,7,8,9,10,11,12]. People are exposed to PFOS through textiles, paper, leather, paints, general cleaning products, carpets and drapery for decades [13]. PFOS accumulates in and produces toxic effects on humans mainly through ingestion of food containing PFOS, inhalation of air and dust containing PFOS and skin contact with PFOS products [14].

PFOS is likely to cause damage to the kidney after it enters the human body [15]. The kidney is one of the main excretory organs for PFOS and its related compounds are excreted through the kidney with no biotransformation [16]. PFOS enters the body and binds to serum proteins and renal transport proteins, but the reabsorption by the kidneys limits the discharge of some PFOS in the urine [10]. Epidemiological investigations have shown that PFOS can lead to kidney damage by reducing the glomerular filtration rate [6,17]. In a mice model, PFOS was found to induce glomerular atrophy and cloudy swelling of the renal tubules, suggesting that PFOS can induce kidney injury [6,18,19]. However, fewer studies have been conducted on animals, and mice appear to be the only animal model in which PFOS has been found to have adverse effects on the kidney. Since PFOS is still produced and applied in many developing countries, it is of great significance to explore the damage of PFOS to the kidney and its relevant mechanisms.

Ferroptosis is a new form of regulated cell death, characterized by the overwhelming iron-dependent accumulation of lethal lipid ROS [20,21,22]. There are several specific markers of ferroptosis, including phospholipid peroxidation, impaired glutathione peroxidase 4 (GPx4) activity and Fe^2+^ accumulation [23]. Downregulation of GPx4, mRNA level for lipid peroxidation remover and upregulation of Acyl-CoA synthetase long-chain family member 4 (ACSL4) are the dominant features of ferroptosis. [24,25]. Iron homeostasis is essential for normal renal function [26], because the tubular cells of the kidney are rich in polyunsaturated fatty acids, which are more sensitive to lipid peroxidation, making them more susceptible to ferroptosis [27]. Inhibition of ferroptosis significantly reduces inflammatory cell accumulation and kidney fibrosis, and targeting ferroptosis attenuates interstitial inflammation and kidney fibrosis [28,29]. However, our current understanding of the effect of PFOS on kidney cell injury remains unclear.

In this study, we chose human proximal tubule epithelial cells (HK-2) as our subject of study and aimed to investigate the role of ferroptosis in the toxic effect of PFOS on HK-2 cells and to elucidate the pathways of PFOS-induced HK2 cell injury and death to provide new insights into the nephrotoxicity of PFOS.

## 2. Materials and Methods

**Figure 1:**
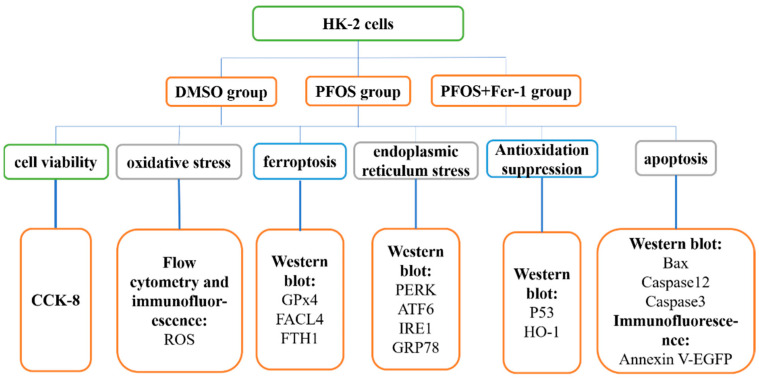
Schematic diagram of materials and methods.

A simple diagram has been drawn to aid in the reading of the Materials and Methods section (Figure 1).

### 2.1. Cell Culture

The human proximal tubular epithelial cell (HK-2) was a gift from Professor Jinyu Liu from the Department of Toxicology, School of Public Health of Jilin University. HK-2 cells were cultured in MEM (Solarbio, Beijing, China) supplemented with 10% fetal bovine serum (FBS, BI. Kibbutz Beit-Haemek, Israel), 1% penicillin-streptomycin (NCM Biotech, Suzhou, China) at 5% CO_2_, 37 °C.

### 2.2. Determination of PFOS Concentration

HK-2 cells were plated into a 96-well plate with each well of 1 × 10^4^ cells. When the confluence of the cells reached 70–80%, PFOS (Swift River Reagent, China) was added to the cells at the concentration of 0 µM, 50 µM, 100 µM, 150 µM, 200 µM and 250 µM, respectively. PFOS and HK-2 cells were cocultured for 12 h. Then, 10 µL of the Enhanced Cell Counting Kit-8 (CCK-8) was added to each well and the cells were cultured with CCK-8 for one or two hours. A microplate reader (PerkinElmer Inc., Boston, MA, USA) was used to determine the absorbance of each well.

### 2.3. Determination of Fer-1 Concentration

HK-2 cells were seeded into a 96-well plate with each well of 1 × 10^4^ cells. When the confluence of the cells was 70–80%, the ferroptosis specific inhibitor Fer-1 (Fer-1, APExBIO, Houston, TX, USA) was added to the cells at the concentration of 0 µM, 1 µM, 2 µM and 5 µM, respectively, and Fer-1 was cocultured with the cells for 2 h. Then, PFOS was added to the cells at the concentration of 200 µM and cultured for 12 h. 10 µL CCK-8 was added to each well and the cells were cultured with CCK-8 for one or two hours. A microplate reader (PerkinElmer Inc., Boston, MA, USA) was used to determine the absorbance of each well.

### 2.4. Cell Treatment

HK-2 cells were plated into flasks. When the confluence of the cells was 60–70%, the cells were assigned to either of the three groups: the control group in which the cells were treated with 0.1%DMSO, the PFOS group in which the cells were treated with 200 µM for 12 h or the PFOS+Fer-1 group in which the cells were treated with Fer-1 (1 µM) for 2 h prior to exposure to PFOS (200 µM) for 12 h.

### 2.5. Measurement of ROS Production by Flowcytometry

From each group, 1 × 10^6^ cells were mixed with fluorescent probe (DCFH-DA, Solarbio, Beijing, China) at the final concentration of DCFH-DA 10 µmol/L and the mixture was cultured at 37 °C for 20 min. The mixture was shaken every 5 min to ensure the complete contact of cells with DCFH-DA. An MEM medium without FBS was used to wash the cells three times to remove DCFH-DA that had not entered into the HK-2 cells. The fluorescence intensity was detected by Cytation 3 Cell Imaging Multi-Mode Reader (BioTek, San Diego, CA, USA). The excitation wavelength and emission wavelength were 488 nm and 525 nm, respectively.

### 2.6. Determination of ROS Production by Immunofluorescence

From each group, 1 × 10^6^ cells were mixed with DCFH-DA (final concentration 10 µmol/L) and they were incubated at 37 °C for 20 min. At a total of 20 min later, the cells were washed with MEM without FBS three times to remove DCFH-DA that had not entered into the cells. Direct observation and photography were conducted by using a laser scanning confocal microscope (BioTek, San Diego, CA, USA); the excitation wavelength and emission wavelength were 488 nm and 525 nm, respectively.

### 2.7. Cell Apoptosis Determined by Immunofluorescence

From each group, 1 × 10^6^ cells were washed with PBS twice. The cell pellets were resuspended using 500 µL of binding buffer and successively mixed with 5 µL of Annexin V-EGFP and 5 µL of propidium iodide. The cells were analyzed on a FACSCalibur (Becton, Dickinson & Co., Franklin Lakes, NJ, USA) following reaction in the dark for 5–15 min. The excitation wavelength and emission wavelength were 488 nm and 530 nm, respectively.

### 2.8. Expression Analysis of Ferroptosis, Endoplasmic Reticulum Stress, Oxidative Stress and Apoptosis Related Protein by Western Blot Analysis

The information on antibodies is as follows: GAPDH (Proteintech, Chicago, IL, USA; 1:2000), ACSL4 (Abcam, Boston, MA, USA; 1:10,000), GPx4 (Abcam, USA; 1:1000), FTH1(Abcam, USA; 1:1000), GRP78 (Proteintech, Chicago, IL, USA; 1:1000), IRE1 (Proteintech, Chicago, IL, USA; 1:1000), ATF6(Proteintech, Chicago, IL, USA; 1:500), PERK (Proteintech, Chicago, IL, USA; 1:500), P53(Proteintech, Chicago, IL, USA; 1:5000), HO-1(Proteintech, Chicago, IL, USA; 1:500), Caspase12 (Proteintech, Chicago, IL, USA; 1:500), Caspase3 (Proteintech, Chicago, IL, USA; 1:1000), Bax(CST, Danvers, MA, USA; 1:1000). The images were obtained by a chemiluminescence imaging analysis system (ECL from Tanon 5200, Shanghai, China) and the band intensities were analyzed by Tanon Gis analytical software (Shanghai, China).

### 2.9. Statistical Analysis

GraphPad Prism version 8.0.2 (GraphPad Software, San Diego, CA, USA, 2018) and IBM SPSS 24.0 (IBM Corp., Armonk, NY, USA, 2016) were used for all analyses and preparations of graphs. All data represented in graphs were expressed as the mean ± SD. ANOVA and LSD methods were used to analyze significant differences; *p*-value was considered significant at *p* < 0.05.

## 3. Results

### 3.1. Determination of PFOS and Fer-1 Concentration

**Figure 2:**
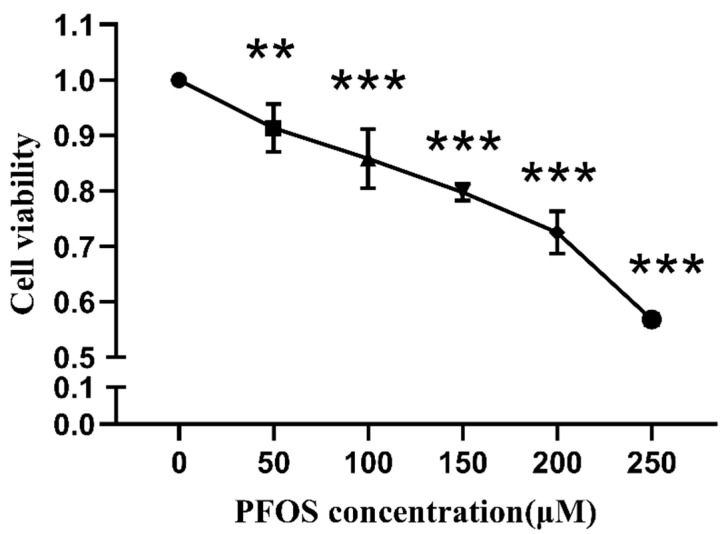
Cell viability after HK-2 cells were exposed to PFOS. HK-2 cells were exposed to PFOS at the concentration of 0 µM, 50 µM, 100 µM, 150 µM, 200 µM and 250 µM for 12 h and CCK-8 was used to detect the cell viability; the results were from three independent experiments. Cell viability was expressed as mean ± SD (n = 3). * vs. control, ** *p* < 0.01, *** *p* < 0.001.

As shown in Figure 2, with the increase in PFOS, the HK-2 cell viability decreased (*p* < 0.05). The cell viability decrease presented a dose–response pattern. When the PFOS concentration was at 200 µM, the cell viability was 72.47%. By examining the cell viability as well as the cell status in combination with the literature data, we selected PFOS at 200 µM for the subsequent experiments. As shown in Figure 2, when the Fer-1 was at 1 µM, the cell viability was 129.52%; by examining the cell viability as well as the cell status in combination with the literature data, we selected Fer-1 at 1 µM for the subsequent experiments.

### 3.2. ROS Production in HK-2 Cells by Exposure to PFOS

**Figure 3:**
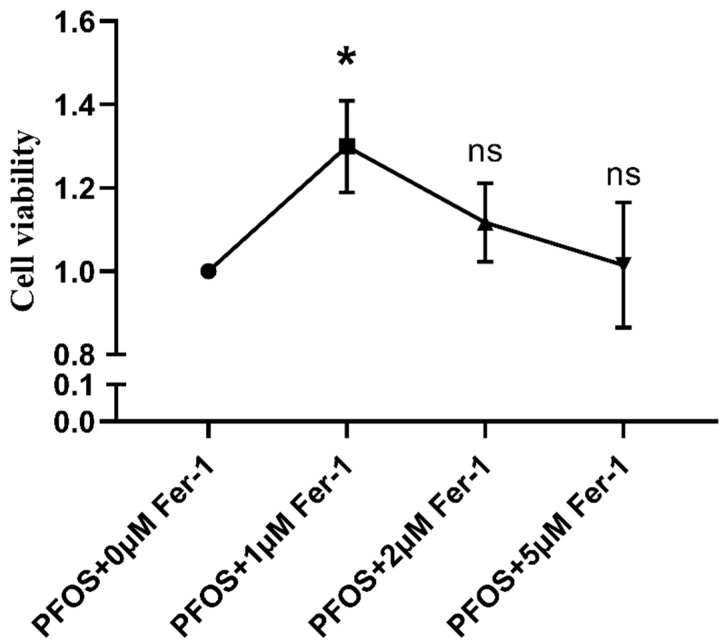
Cell viability after HK-2 cells exposed to PFOS or/and Fer-1. HK-2 cells were exposed to PFOS for 12 h or cultured with Fer-1 for 2 h at the concentration of 0 µM, 1 µM, 2 µM and 5 µM prior to exposure to PFOS and CCK-8 was used to detect the cell viability; the results were from three independent experiments. Cell viability was expressed as mean ± SD (n = 3); * vs. control, *p* < 0.05; ns—no significant difference.

**Figure 4:**
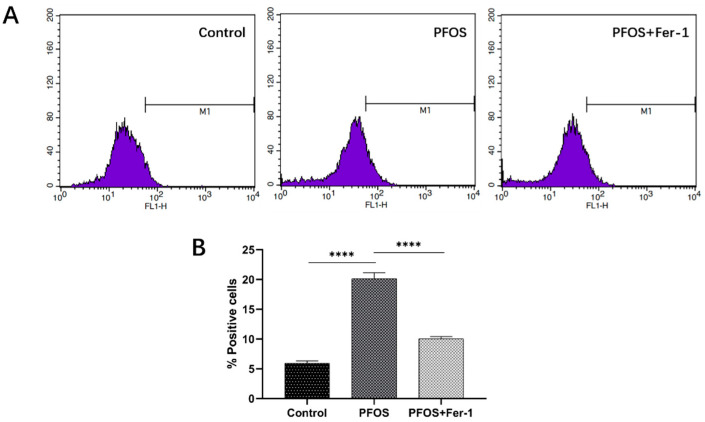
(**A**) ROS production determined by flowcytometry. HK-2 cells were exposed to PFOS at 200 µM for 12 h or/and cultured with Fer-1 at 1 µM for 2 h prior to exposure to PFOS. The excitation wavelength and emission wavelength were 488 nm and 525 nm, respectively. (**B**) Comparison of fluorescence intensity in HK-2 cells exposed to PFOS or/and Fer-1. HK-2 cells were exposed to PFOS at 200 µM for 12 h or/and cultured with Fer-1 at 1 µM for 2 h prior to exposure to PFOS; the results were from three independent experiments. Data were expressed as mean ± SD (n = 3); **** *p* < 0.0001.

**Figure 5:**
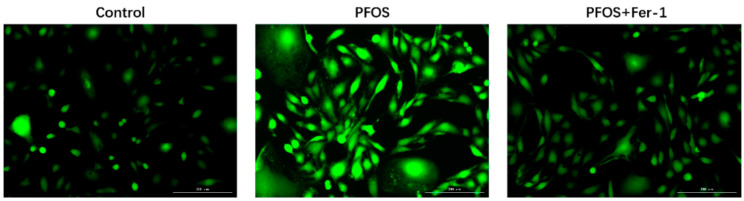
ROS production determined by immunofluorescence in HK-2 cells exposed to PFOS or/and Fer-1. HK-2 cells were exposed to PFOS at 200 µM for 12 h or/and cultured with Fer-1 at 1μM for 2 h prior to exposure to PFOS; the results were from three independent experiments.

**Figure 6:**
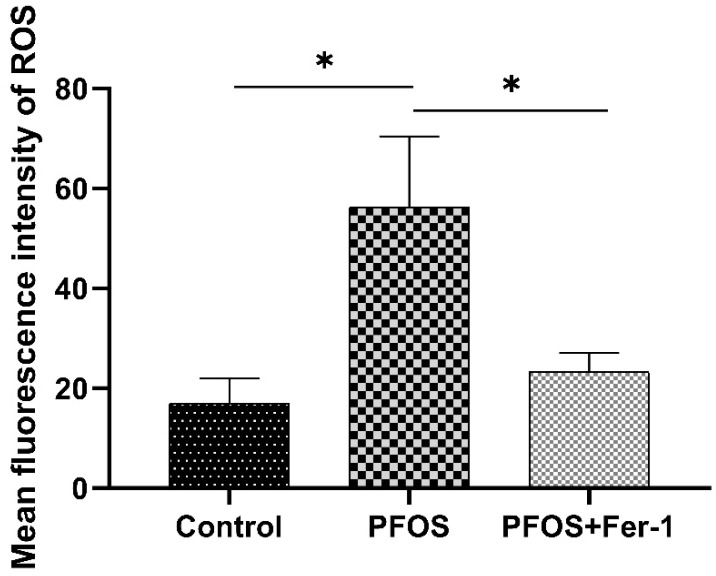
Comparison of immunofluorescence intensity of HK-2 cells exposed to PFOS or/and Fer-1. HK-2 cells were exposed to PFOS at 200 µM for 12 h or/and cultured with Fer-1 at 1 µM for 2 h prior to exposure to PFOS; the results were from three independent experiments. Data were expressed as mean ± SD (n = 3), * *p* < 0.05.

As shown in Figure 3 and Figure 4, PFOS significantly induced the production of ROS in HK-2 cells after the cells were exposed to PFOS for 12 h, and Fer-1 was able to inhibit the ROS production caused by PFOS. The above results indicate that PFOS could induce oxidative stress and Fer-1 could alleviate oxidative stress. Figure 5 and Figure 6 further demonstrate that PFOS could trigger the significant production of ROS and Fer-1 could inhibit the ROS production.

### 3.3. Ferroptosis in HK-2 Cells Induced by PFOS

**Figure 7:**
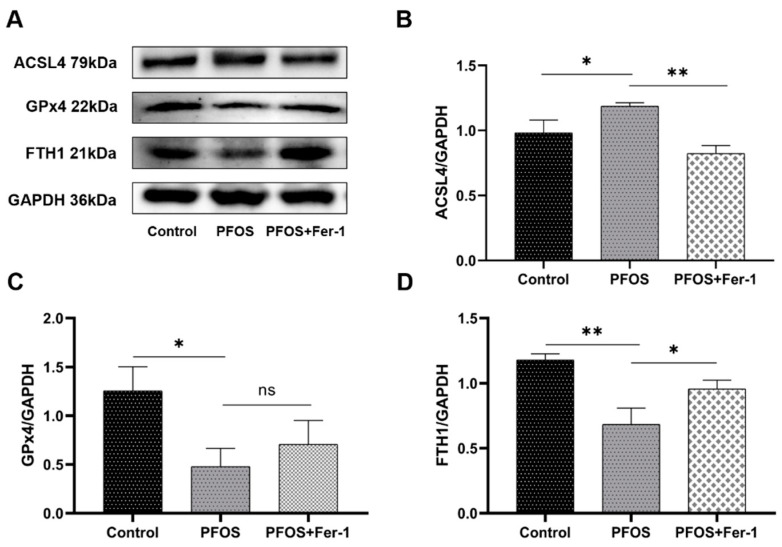
Expression of ACSL4, GPx4 and FTH1 in HK-2 cells exposed to PFOS or/and Fer-1. HK-2 cells were exposed to PFOS at 200 µM for 12 h or/and cultured with Fer-1 at 1 µM for 2 h prior to exposure to PFOS; the results were from three independent experiments. Data were expressed as mean ± SD (n = 3), * *p* < 0.05, ** *p* < 0.01, ns—no significant difference. (**A**) Western blot analysis of ACSL4, GPx4 and FTH1; (**B**) comparison of ACSL4 expression among control, PFOS and PFOS+Fer-1 group cells. (**C**) comparison of GPx4 expression among control, PFOS and PFOS+Fer-1 group cells; (**D**) comparison of FTH1 expression among control, PFOS and PFOS+Fer-1 group cells.

To determine whether PFOS is able to induce ferroptosis, we detected the expression of ferroptosis-related proteins ACSL4, GPx4 and FTH1 in HK-2 cells. As shown in Figure 7, PFOS induced the increased expression of ACSL4 and decreased the expression of GPx4 and FTH1. HK-2 cells treated with Fer-1 prior to exposure to PFOS had significantly decreased the expression of ACSL4 and increased the expression of GPx4 and FTH1 compared with HK-2 cells exposed to PFOS (*p* < 0.05). The results suggest that PFOS could induce ferroptosis and Fer-1 could inhibit the ferroptosis triggered by PFOS and alleviate the damage of HK-2 cells exposed to PFOS.

### 3.4. Endoplasmic Reticulum Stress in HK-2 Cells Caused by PFOS

**Figure 8:**
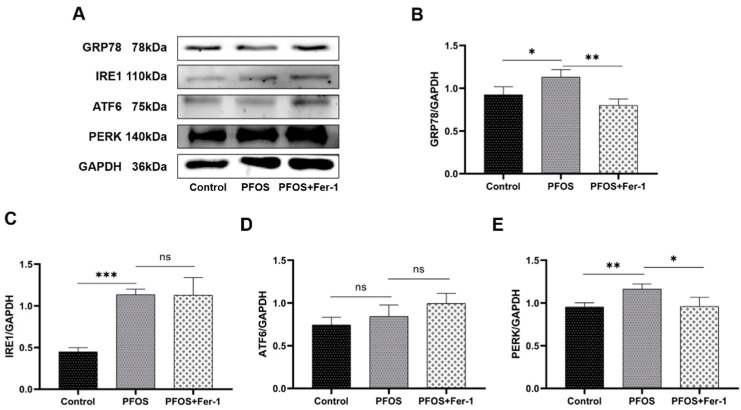
Expression of GRP78, IRE1, ATF6 and PERK in HK-2 cells exposed to PFOS or/and Fer-1. HK-2 cells were exposed to PFOS at 200 µM for 12 h or/and cultured with Fer-1 at 1 µM for 2 h prior to exposure to PFOS; the results were from three independent experiments. Data were expressed as mean ± SD (n = 3), * *p* < 0.05, ** *p* < 0.01, *** *p* < 0.001; ns—no significant difference. (**A**) Western blot of GRP78, IRE1, ATF6 and PERK; (**B**) comparison of GRP78 expression among control, PFOS and PFOS+Fer-1 group cells; (**C**) comparison of IRE1 expression among control, PFOS and PFOS+Fer-1 group cells; (**D**) comparison of ATF6 expression among control, PFOS and PFOS+Fer-1 group cells; (**E**) comparison of PERK expression among control, PFOS and PFOS+Fer-1 group cells.

To determine whether PFOS will induce the endoplasmic reticulum stress in HK-2 cells, we detected the expression of endoplasmic reticulum stress-related proteins GRP78, IRE-1, ATF6 and PERK. As shown in Figure 8, compared with the expression of GRP78, IRE-1 and PERK in HK-2 cells from control group, the expression of GRP78, IRE-1 and PERK in HK-2 cells from PFOS group was significantly increased (*p* < 0.05). Compared with the expression of GPR78 and PERK in HK-2 cells from PFOS group, the expression of GPR78 and PERK in HK-2 cells from PFOS+Fer-1 group was significantly decreased (*p* < 0.05). There was no significant difference in the expression of IRE1 between the PFOS and the PFOS+Fer-1 groups. Moreover, there was no significant difference in the expression of ATF6 in HK-2 cells among the control, the PFOS and the PFOS+Fer-1 groups. These results suggest that PFOS could cause endoplasmic reticulum stress in HK-2 cells and that Fer-1 could alleviate the endoplasmic reticulum stress state caused by PFOS. Therefore, it is indicated that endoplasmic reticulum stress is involved in the onset of PFOS-mediated ferroptosis and the mechanism underlying involves the PERK pathway in endoplasmic reticulum stress.

### 3.5. Antioxidation Suppression by PFOS Exposure

**Figure 9:**
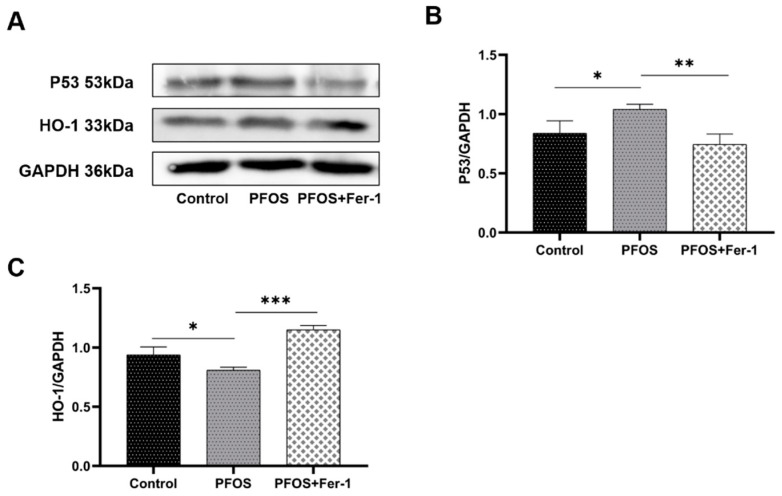
Expression of P53 and HO-1 in HK-2 cells exposed to PFOS or/and Fer-1. HK-2 cells were exposed to PFOS at 200 µM for 12 h or/and cultured with Fer-1 at 1 µM for 2 h prior to exposure to PFOS; the results were from three independent experiments. Data were expressed as mean ± SD (n = 3), * *p* < 0.05, ** *p* < 0.01, *** *p* < 0.001. (**A**) Western blot of P53and HO-1; (**B**) comparison of P53 expression among control, PFOS and PFOS+Fer-1 group cells; (**C**) comparison of HO-1 expression among control, PFOS and PFOS+Fer-1 group cells.

We detected the expression of P53 and HO-1 in HK-2 cells exposed to PFOS to explore the effects of PFOS on the antioxidation. As shown in Figure 9, compared with HK-2 cells from the control group, HK-2 cells from the PFOS group had a significantly increased expression of P53 (*p* < 0.05). As shown in Figure 9C, there was a decline in HO-1 expression in the PFOS group compared with the control group. Compared with the PFOS group, HO-1 expression was increased in the PFOS+Fer-1 group. The above results suggest that PFOS could inhibit the antioxidant status in HK-2 cells and that P53 is involved in this process. Fer-1 improved these indicators and attenuated the suppression of antioxidation, which is involved in the development of ferroptosis.

### 3.6. Apoptosis of HK-2 Cells Caused by PFOS

**Figure 10:**
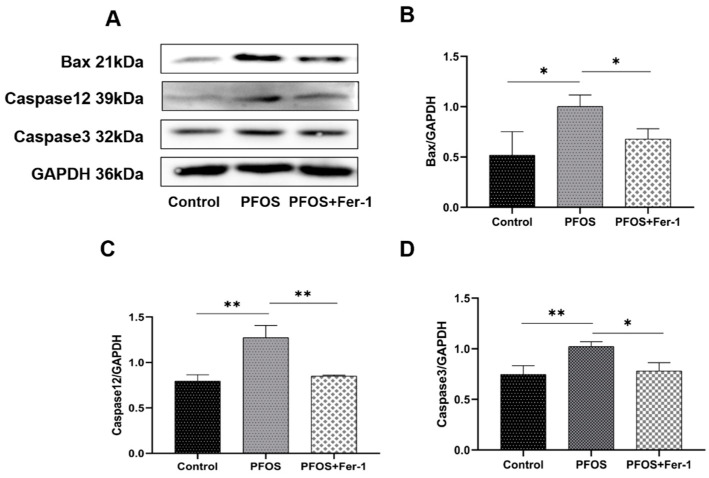
Expression of Bax, Caspase12 and Caspase3 in HK-2 cells exposed to PFOS or/and Fer-1. HK-2 cells were exposed to PFOS at 200 µM for 12 h or/and cultured with Fer-1 at 1 µM for 2 h prior to exposure to PFOS; the results were from three independent experiments. Data were expressed as mean ± SD (n = 3), * *p* < 0.05, ** *p* < 0.01. (**A**) Western blot of Bax, Caspase12 and Caspase3; (**B**) comparison of Bax expression among control, PFOS and PFOS+Fer-1 group cells; (**C**) comparison of Caspase12 expression among control, PFOS and PFOS+Fer-1 group cells; (**D**) comparison of Caspase-3 expression among control, PFOS and PFOS+Fer-1 group cells.

To confirm whether apoptosis is involved in the PFOS-mediated onset of ferroptosis in HK-2 cells, we detected the expression of Bax, Caspase12 and Caspase3 in HK-2 cells by Western blot. As shown in Figure 10B–D, PFOS increased the expression of Bax, Caspase12 and Caspase3, while Fer-1 decreased the expression of Bax, Caspase12 and Caspase3, which indicate that PFOS could lead to endoplasmic reticulum stress-mediated apoptosis in cells and Fer-1 could alleviate the apoptosis of HK-2 cells.

**Figure 11:**
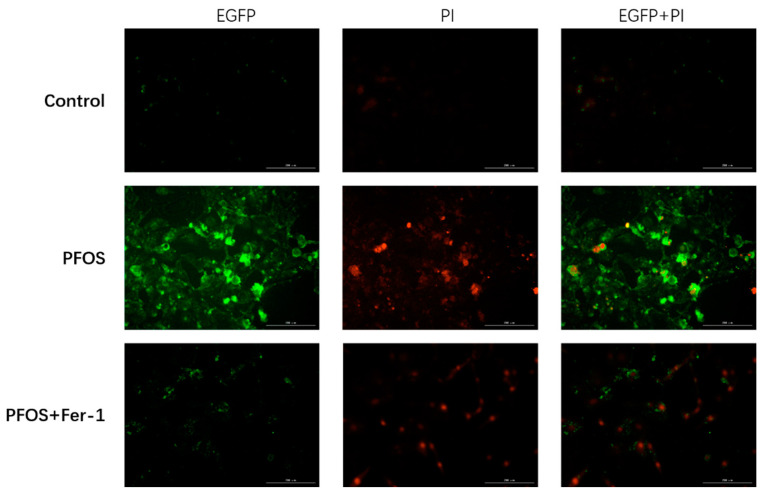
Annexin V-EGFP staining of HK-2 cells exposed to PFOS or/and Fer-1. Normal living cells are not colored by Annexin V-EGFP and PI, cells with early apoptosis are only stained by Annexin V-EGFP and colored green; those stained by PI are dead cells and colored red; necrotic cells and cells with late apoptosis can be stained by Annexin V-EGFP and PI at the same time.

**Figure 12:**
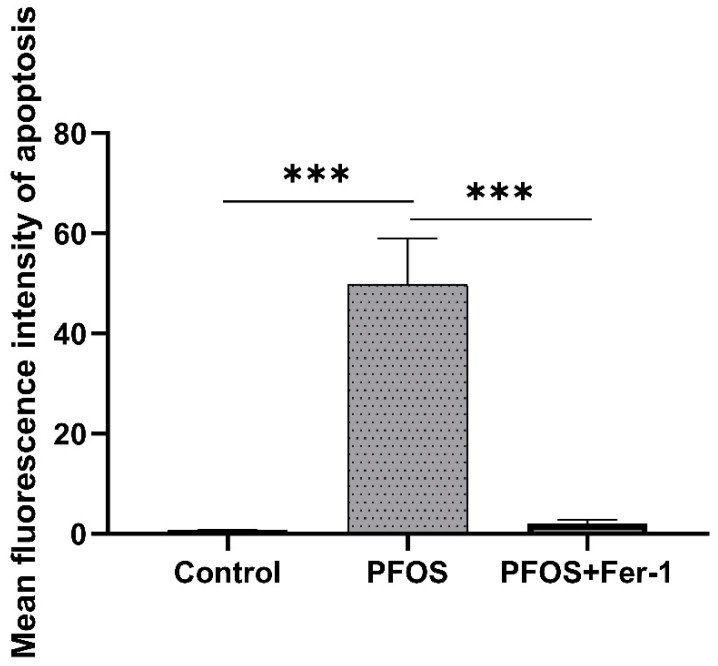
Comparison of apoptosis fluorescence intensity in HK-2 cells exposed to PFOS or/Fer-1. Data are expressed as mean ± SD (n = 3). (*** *p* < 0.001).

As shown in Figure 11 and Figure 12, Annexin V-EGFP was used to detect the apoptosis of cell apoptosis. HK-2 cells exposed to PFOS had more intensive green fluorescence than the control and the PFOS+Fer-1 cells (*p* < 0.05), which indicates that PFOS could trigger the apoptosis of HK-2 cells and Fer-1 could alleviate the apoptosis. In addition, there were significantly more dead cells in the PFOS group than in the other two groups, indicating that PFOS can cause cells death.

## 4. Discussion

PFOS has been listed as one of the 10 pollutants that should receive the most concern worldwide and that poses a tremendous threat to the ecosystems and human health [30,31]. Diet and drinking water are the main routes of exposure to PFOS globally [32,33,34]; other routes of exposure include consumer products, household articles, cleaning products, personal care products and indoor air and dust [35,36]. The kidney is the major organ for excretion of all kinds of toxicants and the toxicants can also do damage to the kidney. A cohort study from Sweden shows that exposure to PFOS is able to increase the risk of developing kidney cancer [37]. In the present study, we explored the effect of PFOS on the human proximal tubular epithelial cell line (HK-2). Our findings show that PFOS can reduce the cell viability of HK-2 cells in a dose-dependent manner and can trigger ferroptosis. To the best of our knowledge, there are no available reports on the ferroptosis caused by PFOS.

Oxidative stress is a risk factor for the development and worsening of kidney diseases [10]. Our study showed that PFOS could induce the development of oxidative stress, which caused damage to HK-2 cells. Our results are in accordance with previous studies showing that PFOS could trigger oxidative stress [38,39,40].

Ferroptosis occurs due to the elevation of the intracellular iron level, which results in the increased level of ROS production and the depletion of antioxidant glutathione (GSH), leading to the death of cells [41]. GPx4 is an antioxidant enzyme that neutralizes lipid peroxides and protects membrane fluidity by using GSH, as a cofactor of GPx4, to protect cells and membranes against peroxidation [42,43]. Inhibiting GPx4 can lead to increased ROS, while overexpression of GPx4 can reduce ROS and subsequently prevent cells from ferroptosis [44]. Our findings demonstrate that PFOS exposure inhibits the expression of GPx4, and when we used ferroptosis inhibitor Fer-1 to treat HK-2 cells prior to exposure to PFOS, the expression of GPx4 was increased, which indicates that PFOS mediated ferroptosis through the GPx4 pathway. ACSL4 is a critical determinant of ferroptosis sensitivity [45]. Compared with ferroptosis-sensitive cells, the expression of ACSL4 was remarkably downregulated in ferroptosis-resistant cells [24]. In our study, PFOS induced the increased expression of ACSL4 in HK-2 cells, and Fer-1 decreased the expression of ACSL4. Ferritin heavy chain 1 (FTH1), a key subunit of ferritin, plays a critical role in maintaining the balance of intracellular iron metabolism and its expression level reflects susceptibility to ferroptosis by in vitro stimuli [20]; our study showed that PFOS could decrease the expression of FTH1. In summary, our study found that GPx4 and FTH1 expression decreased, and ACSL4 expression increased, in HK-2 cells after PFOS exposure, which is a typical marker of ferroptosis [29], indicating that ferroptosis can occur in HK-2 cells after PFOS exposure. Fer-1, a ferroptosis inhibitor [46], can improve the above ferroptosis-related indicators, suggesting that Fer-1 can inhibit the occurrence of PFOS-mediated ferroptosis, which further confirms that ferroptosis occurs in HK-2 cells exposed to PFOS.

To explore whether there are other signaling pathways involved in the onset of iron death in HK-2 cells due to PFOS, we examined endoplasmic reticulum stress, antioxidant mechanisms and parts related to apoptosis. ROS is a key factor that causes protein misfolding in the endoplasmic reticulum and ultimately triggers endoplasmic reticulum stress [47]. Our studies have demonstrated that ROS is involved in the PFOS-mediated onset of iron death in HK-2 cells. In our assay of endoplasmic reticulum stress-related proteins, we showed that the PERK pathway may be involved in the onset of iron death. This suggests that PFOS can lead to endoplasmic reticulum stress in HK-2 cells. The P53 is a tumor suppressor that is stimulated by P53-mediated apoptosis-stimulating proteins in response to DNA damage, induction of apoptosis and ferroptosis [48]. It has been shown that PERK regulates P53 expression, causing P53 to be upregulated [49]. Our results showed that the PERK pathway was significantly upregulated and activated in HK-2 cells in the presence of PFOS and we also found that the expression of P53 was upregulated; therefore, we speculate that PFOS can affect the expression of P53 by activating the PERK pathway in the endoplasmic reticulum stress pathway. Ferrostatin-1(Fer-1) can promote the activation of antioxidant systems and reduces the accumulation of ROS [50]. HO-1 shows cytoprotective effects through antioxidant, anti-apoptotic and anti-inflammatory pathways [51]. There is increasing evidence that under oxidative stress, P53 can inhibit the activation and functional expression of HO-1 by regulating Nrf2 signaling and thereby affecting HO-1 expression [49,50,52,53]. In the presence of Fer-1, P53 expression was downregulated and HO-1 expression was upregulated; this suggests that intracellular antioxidant mechanisms are activated and cells mitigate cellular damage by PFOS by initiating antioxidant defense mechanisms [50]. Our experimental results are only a preliminary speculation that the PERK pathway is associated with the P53-mediated antioxidant pathway. More in-depth mechanisms of endoplasmic reticulum stress and antioxidant pathways, and whether they are cross-linked with the ferroptosis pathways, need to be specifically explored in subsequent studies, which provides a new research direction to explore the mechanism of PFOS toxicity to cells and ferroptosis.

In addition to ferroptosis-related indicators, we also examined some apoptosis-related indicators to explore whether PFOS could mediate apoptosis in HK-2 cells. Although the unfolded protein reaction (UPR) usually helps cell survival by removing unfolded or misfolded proteins, prolonged or excessive endoplasmic reticulum stress may induce activation of Caspase12 [54]. Caspase12 is a specific molecule for endoplasmic reticulum stress-mediated apoptosis and its activation induces activation of the pro-apoptotic protein Bax and fully triggers apoptosis in the caspase pathway [55,56,57]. Our results tentatively suggest that PFOS can lead to endoplasmic reticulum stress-mediated apoptosis in HK-2, but the detailed mechanism needs to be specifically explored in subsequent experiments. Here, we only make a preliminary presentation to more fully assess the cytotoxicity of PFOS.

## 5. Conclusions

In conclusion, our results provide the first evidence that PFOS can lead to the onset of ferroptosis in HK-2 cells and we found that PFOS could reduce HK-2 cell viability in a dose-dependent manner, produce toxic effects on cells and induce ferroptosis and apoptosis in HK-2 cells. In addition, we found that the endoplasmic reticulum stress pathway and the P53-mediated antioxidant pathway are involved in the toxic damage to HK-2 cells by PFOS. These findings provide a new direction to study the mechanism of PFOS toxicity and its damage to the kidney.

## Data Availability

Not applicable.

## References

[1] Leung S., Shukla P., Chen D., Eftekhari E., An H., Zare F., Ghasemi N., Zhang D., Nguyen N.T., Li Q. (2022). Emerging technologies for PFOS/PFOA degradation and removal: A review. Sci. Total Environ..

[2] Savvaides T., Koelmel J.P., Zhou Y., Lin E.Z., Stelben P., Aristizabal-Henao J.J., Bowden J.A., Godri Pollitt K.J. (2021). Prevalence and Implications of Per- and Polyfluoroalkyl Substances (PFAS) in Settled Dust. Curr. Environ. Health Rep..

[3] Baabish A., Sobhanei S., Fiedler H. (2021). Priority perfluoroalkyl substances in surface waters—A snapshot survey from 22 developing countries. Chemosphere.

[4] Olsen G.W., Burris J.M., Ehresman D.J., Froehlich J.W., Seacat A.M., Butenhoff J.L., Zobel L.R. (2007). Half-life of serum elimination of perfluorooctanesulfonate, perfluorohexanesulfonate, and perfluorooctanoate in retired fluorochemical production workers. Environ. Health Perspect..

[5] Mokra K. (2021). Endocrine disruptor potential of short- and long-chain perfluoroalkyl substances (PFASs)—A synthesis of current knowledge with proposal of molecular mechanism. Int. J. Mol. Sci..

[6] Yang C., Lee H.K., Zhang Y., Jiang L.L., Chen Z.F., Chung A., Cai Z. (2019). In Situ detection and imaging of PFOS in mouse kidney by matrix-assisted laser desorption/ionization imaging mass spectrometry. Anal. Chem..

[7] Midgett K., Peden-Adams M.M., Gilkeson G.S., Kamen D.L. (2015). In vitro evaluation of the effects of perfluorooctanesulfonic acid (PFOS) and perfluorooctanoic acid (PFOA) on IL-2 production in human T-cells. J. Appl. Toxicol..

[8] Zhang L., Duan X., Sun W., Sun H. (2020). Perfluorooctane sulfonate acute exposure stimulates insulin secretion via GPR40 pathway. Sci. Total Environ..

[9] Chen J., Miao Y., Gao Q., Cui Z., Xiong B. (2021). Exposure to perfluorooctane sulfonate in vitro perturbs the quality of porcine oocytes via induction of apoptosis. Environ. Pollut..

[10] Umar Ijaz M., Rauf A., Mustafa S., Ahmed H., Ashraf A., Al-Ghanim K., Swamy Mruthinti S., Mahboob S. (2022). Pachypodol attenuates Perfluorooctane sulphonate-induced testicular damage by reducing oxidative stress. Saudi J. Biol. Sci..

[11] Mao B., Mruk D., Lian Q., Ge R., Li C., Silvestrini B., Cheng C.Y. (2018). Mechanistic Insights into PFOS-mediated sertoli cell injury. Trends Mol. Med..

[12] Coperchini F., Awwad O., Rotondi M., Santini F., Imbriani M., Chiovato L. (2017). Thyroid disruption by perfluorooctane sulfonate (PFOS) and perfluorooctanoate (PFOA). J. Endocrinol. Investig..

[13] Lin P.D., Cardenas A., Hauser R., Gold D.R., Kleinman K.P., Hivert M.F., Calafat A.M., Webster T.F., Horton E.S., Oken E. (2021). Per- and polyfluoroalkyl substances and kidney function: Follow-up results from the diabetes prevention program trial. Environ. Int..

[14] Poothong S., Papadopoulou E., Padilla-Sánchez J.A., Thomsen C., Haug L.S. (2020). Multiple pathways of human exposure to poly- and perfluoroalkyl substances (PFASs): From external exposure to human blood. Environ. Int..

[15] Surma M., Hliwa P., Sznajder-Katarzyńska K., Wiczkowski W., Topolska J., Zieliński H. (2021). Perfluoroalkyl Substance contamination levels of pike (*Esox lucius* L.) and roach (*Rutilus rutilus* L.) from selected Masurian lakes in Eastern Europe. Environ. Toxicol. Chem..

[16] Bursian S.J., Link J.E., McCarty M., Harr K., Roberts J., Simcik M.F. (2021). Dietary Exposure of Japanese Quail (Coturnix japonica) to Perfluorooctane Sulfonate (PFOS) and a Legacy Aqueous Film-Forming Foam (AFFF) Containing PFOS: Effects on Reproduction and Chick Survivability and Growth. Environ. Toxicol. Chem..

[17] Kataria A., Trachtman H., Malaga-Dieguez L., Trasande L. (2015). Association between perfluoroalkyl acids and kidney function in a cross-sectional study of adolescents. Environ. Health Glob. Access Sci. Source.

[18] Wang L.Q., Liu T., Yang S., Sun L., Zhao Z.Y., Li L.Y., She Y.C., Zheng Y.Y., Ye X.Y., Bao Q. (2021). Perfluoroalkyl substance pollutants activate the innate immune system through the AIM2 inflammasome. Nat. Commun..

[19] Chen Y., Jiang L., Zhang R., Shi Z., Xie C., Hong Y., Wang J., Cai Z. (2022). Spatially revealed perfluorooctane sulfonate-induced nephrotoxicity in mouse kidney using atmospheric pressure MALDI mass spectrometry imaging. Sci. Total Environ..

[20] Dixon S.J., Lemberg K.M., Lamprecht M.R., Skouta R., Zaitsev E.M., Gleason C.E., Patel D.N., Bauer A.J., Cantley A.M., Yang W.S. (2012). Ferroptosis: An iron-dependent form of nonapoptotic cell death. Cell.

[21] Cai Y., Yang Z. (2021). Ferroptosis and its role in epilepsy. Front. Cell. Neurosci..

[22] Tang D., Chen X., Kang R., Kroemer G. (2021). Ferroptosis: Molecular mechanisms and health implications. Cell Res..

[23] Yuan H., Li X., Zhang X., Kang R., Tang D. (2016). Identification of ACSL4 as a biomarker and contributor of ferroptosis. Biochem. Biophys. Res. Commun..

[24] Yang W.S., SriRamaratnam R., Welsch M.E., Shimada K., Skouta R., Viswanathan V.S., Cheah J.H., Clemons P.A., Shamji A.F., Clish C.B. (2014). Regulation of ferroptotic cancer cell death by GPX4. Cell.

[25] Jin T., Chen C. (2022). Umbelliferone delays the progression of diabetic nephropathy by inhibiting ferroptosis through activation of the Nrf-2/HO-1 pathway. Food Chem. Toxicol..

[26] Dutta R.K., Kondeti V.K., Sharma I., Chandel N.S., Quaggin S.E., Kanwar Y.S. (2017). Beneficial effects of myo-inositol oxygenase deficiency in cisplatin-induced AKI. J. Am. Soc. Nephrol. JASN.

[27] Zhou L., Xue X., Hou Q., Dai C. (2021). Targeting ferroptosis attenuates interstitial inflammation and kidney fibrosis. Kidney Dis..

[28] Yao W., Liao H., Pang M., Pan L., Guan Y., Huang X., Hei Z., Luo C., Ge M. (2022). Inhibition of the NADPH oxidase pathway reduces ferroptosis during septic renal injury in diabetic mice. Oxidative Med. Cell. Longev..

[29] Yang Y., Zhang X., Jiang J., Han J., Li W., Li X., Yee Leung K.M., Snyder S.A., Alvarez P. (2022). Which micropollutants in water environments deserve more attention globally?. Environ. Sci. Technol..

[30] Shi B., Wang T., Yang H., Zhou Y., Bi R., Yang L., Yoon S.J., Kim T., Khim J.S. (2021). Perfluoroalkyl acids in rapidly developing coastal areas of China and South Korea: Spatiotemporal variation and source apportionment. Sci. Total Environ..

[31] Greaves A.K., Letcher R.J., Sonne C., Dietz R., Born E.W. (2012). Tissue-specific concentrations and patterns of perfluoroalkyl carboxylates and sulfonates in East Greenland polar bears. Environ. Sci. Technol..

[32] Domingo J.L., Nadal M. (2019). Human exposure to per- and polyfluoroalkyl substances (PFAS) through drinking water: A review of the recent scientific literature. Environ. Res..

[33] Hu X.C., Andrews D.Q., Lindstrom A.B., Bruton T.A., Schaider L.A., Grandjean P., Lohmann R., Carignan C.C., Blum A., Balan S.A. (2016). Detection of poly- and perfluoroalkyl substances (PFASs) in U.S. drinking water linked to industrial sites, military fire training areas, and wastewater treatment plants. Environ. Sci. Technol. Lett..

[34] DeLuca N.M., Minucci J.M., Mullikin A., Slover R., Cohen Hubal E.A. (2022). Human exposure pathways to poly- and perfluoroalkyl substances (PFAS) from indoor media: A systematic review. Environ. Int..

[35] Poothong S., Padilla-Sánchez J.A., Papadopoulou E., Giovanoulis G., Thomsen C., Haug L.S. (2019). Hand wipes: A useful tool for assessing human exposure to poly- and perfluoroalkyl substances (PFASs) through hand-to-mouth and dermal contacts. Environ. Sci. Technol..

[36] Li H., Hammarstrand S., Midberg B., Xu Y., Li Y., Olsson D.S., Fletcher T., Jakobsson K., Andersson E.M. (2022). Cancer incidence in a Swedish cohort with high exposure to perfluoroalkyl substances in drinking water. Environ. Res..

[37] Basile D.P., Bonventre J.V., Mehta R., Nangaku M., Unwin R., Rosner M.H., Kellum J.A., Ronco C., ADQI XIII Work Group (2016). Progression after AKI: Understanding maladaptive repair processes to predict and identify therapeutic treatments. J. Am. Soc. Nephrol. JASN.

[38] Qin W., Ren X., Zhao L., Guo L. (2022). Exposure to perfluorooctane sulfonate reduced cell viability and insulin release capacity of β cells. J. Environ. Sci..

[39] Bi C., Junaid M., Liu Y., Guo W., Jiang X., Pan B., Li Z., Xu N. (2022). Graphene oxide chronic exposure enhanced perfluorooctane sulfonate mediated toxicity through oxidative stress generation in freshwater clam Corbicula fluminea. Chemosphere.

[40] Stockwell B.R., Friedmann Angeli J.P., Bayir H., Bush A.I., Conrad M., Dixon S.J., Fulda S., Gascón S., Hatzios S.K., Kagan V.E. (2017). Ferroptosis: A Regulated Cell Death Nexus Linking Metabolism, Redox Biology, and Disease. Cell.

[41] Su L.J., Zhang J.H., Gomez H., Murugan R., Hong X., Xu D., Jiang F., Peng Z.Y. (2019). Reactive oxygen species-induced lipid peroxidation in apoptosis, autophagy, and ferroptosis. Oxidative Med. Cell. Longev..

[42] Conrad M., Kagan V.E., Bayir H., Pagnussat G.C., Head B., Traber M.G., Stockwell B.R. (2018). Regulation of lipid peroxidation and ferroptosis in diverse species. Genes Dev..

[43] Kinowaki Y., Kurata M., Ishibashi S., Ikeda M., Tatsuzawa A., Yamamoto M., Miura O., Kitagawa M., Yamamoto K. (2018). Glutathione peroxidase 4 overexpression inhibits ROS-induced cell death in diffuse large B-cell lymphoma. Lab. Investig..

[44] Doll S., Proneth B., Tyurina Y.Y., Panzilius E., Kobayashi S., Ingold I., Irmler M., Beckers J., Aichler M., Walch A. (2017). ACSL4 dictates ferroptosis sensitivity by shaping cellular lipid composition. Nat. Chem. Biol..

[45] Fang Y., Chen X., Tan Q., Zhou H., Xu J., Gu Q. (2021). Inhibiting Ferroptosis through Disrupting the NCOA4-FTH1 Interaction: A New Mechanism of Action. ACS Cent. Sci..

[46] Jiang L., Kon N., Li T., Wang S.J., Su T., Hibshoosh H., Baer R., Gu W. (2015). Ferroptosis as a p53-mediated activity during tumour suppression. Nature.

[47] Hu Q., Zheng J., Xu X.N., Gu C., Li W. (2022). Uranium induces kidney cells apoptosis via reactive oxygen species generation, endoplasmic reticulum stress and inhibition of PI3K/AKT/mTOR signaling in culture. Environ. Toxicol..

[48] Zheng X., Liu B., Liu X., Li P., Zhang P., Ye F., Zhao T., Kuang Y., Chen W., Jin X. (2022). PERK Regulates the sensitivity of hepatocellular carcinoma cells to High-LET carbon ions via either apoptosis or ferroptosis. J. Cancer.

[49] Kalo E., Kogan-Sakin I., Solomon H., Bar-Nathan E., Shay M., Shetzer Y., Dekel E., Goldfinger N., Buganim Y., Stambolsky P. (2012). Mutant p53R273H attenuates the expression of phase 2 detoxifying enzymes and promotes the survival of cells with high levels of reactive oxygen species. J. Cell Sci..

[50] Jiao Y., Wang S., Jiang L., Sun X., Li J., Liu X., Yao X., Zhang C., Wang N., Deng H. (2022). 2-undecanone protects against fine particles-induced heart inflammation via modulating Nrf2/HO-1 and NF-κB pathways. Environ. Toxicol..

[51] Wegiel B., Nemeth Z., Correa-Costa M., Bulmer A.C., Otterbein L.E. (2014). Heme oxygenase-1: A metabolic nike. Antioxid. Redox Signal..

[52] Liu X., Dilxat T., Shi Q., Qiu T., Lin J. (2022). The combination of nicotinamide mononucleotide and lycopene prevents cognitive impairment and attenuates oxidative damage in D-galactose induced aging models via Keap1-Nrf2 signaling. Gene.

[53] Zhang X., Ding M., Zhu P., Huang H., Zhuang Q., Shen J., Cai Y., Zhao M., He Q. (2019). New Insights into the Nrf-2/HO-1 Signaling Axis and Its Application in Pediatric Respiratory Diseases. Oxidative Med. Cell. Longev..

[54] Wang X., An Y., Jiao W., Zhang Z., Han H., Gu X., Teng X. (2018). Selenium Protects against Lead-induced Apoptosis via Endoplasmic Reticulum Stress in Chicken Kidneys. Biol. Trace Elem. Res..

[55] Guo Y., Hao D., Hu H. (2021). High doses of dexamethasone induce endoplasmic reticulum stress-mediated apoptosis by promoting calcium ion influx-dependent CHOP expression in osteoblasts. Mol. Biol. Rep..

[56] Baek A.R., Hong J., Song K.S., Jang A.S., Kim D.J., Chin S.S., Park S.W. (2020). Spermidine attenuates bleomycin-induced lung fibrosis by inducing autophagy and inhibiting endoplasmic reticulum stress (ERS)-induced cell death in mice. Exp. Mol. Med..

[57] Wang T., Feng X., Li L., Luo J., Liu X., Zheng J., Fan X., Liu Y., Xu X., Zhou G. (2022). Effects of quercetin on tenderness, apoptotic and autophagy signalling in chickens during post-mortem ageing. Food Chem..

